# Patient-Derived Cancer Models

**DOI:** 10.3390/cancers12123779

**Published:** 2020-12-15

**Authors:** Maria Flavia Di Renzo, Simona Corso

**Affiliations:** 1 Department of Oncology, University of Torino, 10124 Torino, Italy; 2 Candiolo Cancer Institute, FPO-IRCCS, Candiolo, 10060 Torino, Italy

For many decades, basic and preclinical cancer research has been based on the use of established, commercially available cell lines, originally derived from patients’ samples but adapted to grow indefinitely in artificial culture conditions, and on xenograft models developed by injection of these cells in immunocompromised animals. These models have been extremely useful for shedding light on cancer cell biology; however, in a number of cases, they have proved to be unsuitable for biomarker discovery, drug screening, and therapeutic preclinical testing [1]. 

The effort to find preclinical models able to better predict the clinical outcome led to the generation of patient-derived cancer models, obtained either by propagating fresh tumor tissues in experimental animals, e.g., patient-derived xenograft models (PDXs); deriving 3D structures from human cancer tissues, i.e., organoids; or maintaining tumor cells under in vitro 2D tissue culture conditions for a short period. 

PDXs have been successfully derived from a variety of solid or hematologic primary and metastatic cancers [2,3] by applying different procedures. Cancer samples can be subcutaneously or orthotopically xenografted to recapitulate microenvironmental interactions within patients. In particular, acute leukemias and other bone-marrow-resident disorders readily undergo orthotopic engraftment after tail-vein or intraosseous injection. PDXs well recapitulate the genetic, transcriptional, and histological features of the original tumors [4]. The genetic stability of the PDX model through successive mouse-to-mouse passages in vivo has been questioned [5], but more recent works have shown their genomic fidelity with respect to the originating patient tumors [6]. Moreover, they proved to have high predictive power in biomarker discovery and drug testing, for both molecular compounds and chemotherapy [7,8,9]. Compared to genetically engineered mouse models (GEMMs), which similarly proved to successfully predict clinical efficacy [10], PDX establishment is simpler and faster. However, the main advantage of GEMMs is their proficient immune system. The next-generation forms of PDXs are humanized models, in which selected immune components are introduced in mice in an effort to generate a (partially) competent human immune system [11,12]. 

Concerning in vitro derivatives, several groups have elaborated specific protocols for cell isolation and 3D or 2D cultures from various tumor types, and their genotype-driven responses have been confirmed in vivo in matched PDXs [13,14]. For their simplicity and low cost, 2D models are widely used for high-throughput screenings, even if they are usually limited by low proliferative capacity in culture. Three-dimensional cultures better mimic the physical features and the architecture of the original (solid) tumors, even if they lack the stroma component [15]. Novel techniques, however, have been recently proposed in order to obtain cancer organoids containing fibroblasts and immune cells [16,17].

All these patient-derived experimental models should be considered complementary and not alternative, as every model system is imperfect and suitable in its own way. 

This Special Issue aims at improving our understanding of the possibilities and limitations of patient-derived cancer models by including works not only from investigators using these models but also from those who are engaged in developing novel models, e.g., those for cancer immunology studies.
